# Fork to farm: reverse engineering a food system

**DOI:** 10.1098/rstb.2024.0158

**Published:** 2025-09-18

**Authors:** John Ingram, David Barling, Natasha Bayes, Julian Cottee, Angela Dickinson, Charlotte Hardman, Eric Holub, Katie Jones, Craig Leadley, Rosanne Maguire, Lucy Michaels, Gerald Midgley, Raghav Rajagopalan, Jing Zhang, Monika Zurek

**Affiliations:** ^1^ Environmental Change Institute, SoGE, University of Oxford, Oxford OX1 3QY, UK; ^2^ Department of Agricultural Economics, Stellenbosch University, 7600 Stellenbosch, South Africa; ^3^ Centre for Systems Studies, University of Birmingham, Birmingham B15 2TT, UK; ^4^ Centre for Agriculture, Food and Environmental Management Research, University of Hertfordshire, Hatfield AL10 9AB, UK; ^5^ Department of Psychology, University of Liverpool, Liverpool L69 7ZX, UK; ^6^ 3Keel, Oxford OX1 2BN, UK; ^7^ Centre for Research in Public Health and Community Care, University of Hertfordshire, Hatfield AL10 9AB, UK; ^8^ Warwick Crop Centre, University of Warwick, Coventry, UK; ^9^ Campden BRI, Chipping Campden, Gloucestershire GL55 6LD, UK; ^10^ School of Life Sciences, Warwick Crop Centre, University of Warwick, Coventry, UK; ^11^ Food and Environmental Management Research, University of Hertfordshire, Hatfield AL10 9AB, UK; ^12^ Birmingham Leadership Institute, University of Birmingham, Birmingham B15 2TT, UK; ^13^ Department of Informatics, Linnaeus University, 392 31 Kalmar, Sweden

**Keywords:** beans, systems thinking, systemic innovation, dietary change, school meals, food policy

## Abstract

Using two new common (*Phaseolus*) dry bean varieties developed for UK growing conditions, the BeanMeals project explored how to pursue ‘fork to farm’ systemic innovation in the food system to transform institutional catering and home-cooking towards healthier diets with lower environmental impact, while also enhancing local and national enterprise. Action research, underpinned by a new systems thinking framework, centred on six primary schools and ten households in Leicester and Leicestershire (UK), set against a review of city-, county- and national-level school food policies. Three demand scenarios were developed, based on increasing UK average daily consumption from 8.5 g to either 17, 34 or 50 g, together with three enterprise opportunities (‘Community Enterprise’, ‘Artisanal Entrepreneurs’ and ‘Food Giants’), to satisfy these demands in different ways. The benefits and trade-offs of scaling UK beans were analysed, including assessments of overall benefits to health, benefits to the environment (which depend on the methods of land conversion and weed management used), and economic benefits (which depend on the scaling method employed).

This article is part of the theme issue ‘Transforming terrestrial food systems for human and planetary health’.

## 1. Introduction

### (a) Systemic innovation to transform the bean food system

A key challenge for the UK food system is how to move towards healthier diets with lower environmental impact while also enhancing local and national enterprise, including farmer incomes. BeanMeals was an action research project designed to address this challenge by exploring systemic innovation in the food system. This was aimed at transforming institutional catering and home-cooking by using UK-grown beans as healthier ingredients, and by changing public procurement practices to use more-local products. An action research approach was appropriate for this because it can facilitate change while simultaneously researching what the change process involves [1].

**Figure 1.**
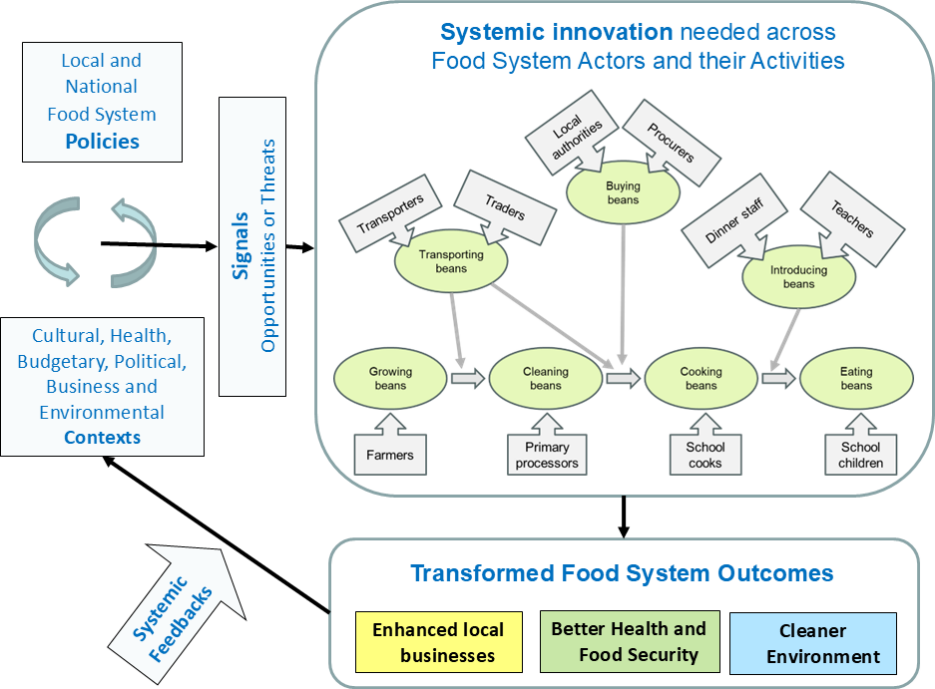
Transforming the bean food system outcomes. The core bean system activities (ellipses) are each undertaken by a specialized actor (arrowed boxes). To transform the outcomes in a balanced way needs ‘systemic innovation’ (see text). Once transformed, the outcomes feed back to alter the contexts within which the bean system operates. Such alternations affect the signals influencing the behaviour of the bean system actors, and hence further transform the bean system outcomes. This therefore calls for re-assessing the public and private policies that, together with the contexts, deliver the signals that influence the actors.

To transform food systems towards better health, environmental and enterprise outcomes requires understanding how to transform the wider bean system, not just its individual activities. This is a key aspect of ‘systemic innovation’: collaboration between multiple actors, taking account of the wider system that the innovation is, or will be, embedded in [2]. To this end, BeanMeals used food systems conceptual frameworks [3] and systems approaches [4] to describe and map the activities, purposes, values, conflicts and boundary judgements of bean supply chain actors and related stakeholders (figure 1), as well as to design and evaluate potential systemic innovations.

BeanMeals was centred on Leicester and Leicestershire in the UK. In addition to addressing pressing malnutrition problems accentuated by COVID-19, this city and county combination provided an ideal testbed for scaling outputs on policy and practice across other regions with challenging health, environmental and economic concerns. This is because it included (i) poorer city wards and rural areas with unhealthy food consumption by children; (ii) extensive ethnic and cultural diversity; and (iii) well defined jurisdictions of nested city–county–region policy processes, which were already linked to the national level to facilitate scaling.

### (b) Why ‘fork to farm’?

By starting the study with the meal and working backwards through the supply chain to the farmers, BeanMeals was based on a ‘fork-to-farm’ approach, reversing the conventional ‘farm-to-fork’, productionist paradigm. The ‘fork’ represents the end point of what a variety of stakeholders want in terms of health, economic and environmental outcomes. ‘Fork to farm’ should not be misinterpreted as a consumption-driven system in the usual business sense—starting with the desires of potential customers and then tailoring products to meet them. A range of health, economic and environmental outcomes need to be considered, whether or not any given set of consumers values or is even aware of them.

### (c) Using UK-grown beans

Most beans consumed in the UK are navy beans canned in tomato sauce. These are imported from North America, as varieties suitable for canning have not been suitable to grow commercially under UK conditions. To research systemic innovation, BeanMeals used two new varieties of UK-registered *Phaseolus* beans (collectively called URBeans), including ‘Capulet’ (with a similar appearance to imported navy beans) and ‘Godiva’ (a novel, blonde-coloured bean). Both have been developed at the University of Warwick using conventional breeding to adapt them to UK conditions [5].

BeanMeals used these pre-commercial varieties in its action research, looking at how to help transform UK food systems away from high fat–sugar–salt (HFSS) diets by developing affordable and acceptable alternatives for a range of cuisines to improve health outcomes. Growing beans also has environmental benefits: as legumes, they can obtain nitrogen fixed from the atmosphere by symbiotic bacteria, lowering requirements for nitrogen fertilizer and thereby reducing emissions of CO_2_ and N_2_O associated with fertilizer manufacturing [6,7].

The purpose of this paper is to present insights from the BeanMeals project using a systemic innovation frame.

## 2. What is systemic innovation?

The term ‘innovation’ originally came from industry, but is now used much more widely by businesses, governments, non-government organizations and communities to describe beneficial changes to products, services, policies and activities. When the word ‘systemic’ is put in front of ‘innovation’, it generally means taking account of the wider system, so the innovation works in the context of that system to deliver desired outcomes while minimizing undesired side-effects.

Systems have interrelated parts, giving rise to emergent properties that can only be understood in terms of the whole system, not any one part in isolation. In food systems, these emergent properties are often referred to as the food system outcomes, whether intended (e.g. commodity production and the consumption of meals) or unintended (e.g. climate change and biodiversity reduction). The system boundaries, relationships and emergent properties are inevitably seen differently from different stakeholder perspectives. In complex systems, nobody can see the whole: all perspectives are partial, as everybody has their own position and role within the system, and they filter what they pay attention to according to their expectations, purposes and values, informed by their past experiences [8,9].

The term ‘systemic innovation’ has been understood in five different ways [10], but instead of seeing these as competing definitions, we can reframe them as different foci for innovation, all of which are relevant to food systems:

(1) *Products*, where technological innovations by different organizations are joined up to create something new and profitable that could not have been achieved by a single organization. For new products incorporating UK beans to reach the market, dovetailed innovations must happen across the supply chain.
(2) *Policies*, which are put in place by governments to facilitate product innovation. To promote innovation with UK beans, policy coordination across the domains of health, environment and the economy would be useful.
(3) *Sustainability*, where the focus is transformational change that will create ripple effects throughout the system, particularly to realize longer-term environmental and socioeconomic outcomes. This is about making a key change that will enable other things to change around it, such as shifting the balance of consumption from meat to UK bean protein, which would reduce the UK’s contribution to climate change in addition to providing new enterprise opportunities and health benefits [11].
(4) *Value-capture* in networks and social ecosystems, where the focus is not just products and financial benefits, but the realization of value (of whatever kind) by diverse participants, including consumers and other non-industrial participants who are not normally thought of as partners in innovation. An example is the innovation of health education in schools, with students learning about beans, which could have both public health value (from improved nutrition) and economic value (longer-term expansion of the market for UK beans).
(5) *Systems thinking*, which is the use of systems concepts and methods to better understand the wider systemic requirements and effects of existing and future innovations [12,13]. Essentially, *systems thinking supports critical thinking about innovation* [10]. This is because it asks people to consider, and *rethink*, key interrelationships (e.g. what human and non-human relationships within the bean system matter?), boundaries (e.g. what elements of the bean system are either important or irrelevant, who is a stakeholder, and is there marginalization that needs to be countered?), emergent properties (e.g. what outcomes matter, recognizing that, in the bean system, multiple outcomes may be in tension?) and perspectives (e.g. who has different viewpoints on the necessary relationships, boundaries and outcomes of the bean system, and how do we learn from them, dealing constructively with conflict along the way?).

Midgley & Lindhult [10] argue that the fifth focus of systemic innovation (systems thinking) is the most useful, because it can enhance all the others. BeanMeals therefore defined systemic innovation as *the use of systems thinking to support innovation processes*.

### (a) Three layers of systems thinking for systemic innovation

A new framework was devised to support the project, recognizing three different ‘layers’ of systems thinking, with each building on the one before. The first layer offers a relatively simple representation of a system, using some basic systems concepts (boundaries, relationships, emergent properties and perspectives). The second layer builds on it by showing how these concepts can be abstracted out of that simple representation for use when practising some key skills for rethinking the system: e.g. shifting the boundary, mapping interconnections, looking for new emergent properties or seeing the system from different perspectives. There are no formal methods offered in layers one and two: the foci are just concepts and thinking skills. The third layer, built on top of the last two, codifies these concepts and skills into a substantial set of specialist systems methodologies and methods to support systemic innovation. While many of these specialist methodologies were originally developed independently from one another over the second half of the twentieth century, twenty-first-century research has demonstrated that they can be integrated together into a flexible practice [8,14]. This involves exploring the context and creatively designing innovation initiatives using a mix of methods drawn from the set of specialized methodologies (with new ones being developed when required). More detail on each of the three layers is provided below.

The first layer is the least complex and is relatively easily learned. It just involves using the idea of a system to think about what gives rise to an innovation, or could give rise to one in future. The system is seen as a real-world collection of people, resources, non-human elements and activities interacting to produce outcomes [2]. However, this does not imply perfect objectivity or comprehensive knowledge: all the perspectives of participants in complex systems (including those of researchers) are inevitably partial, because they are informed by the expectations, purposes, values and past experiences that those participants bring to their observations, communications and actions [8,9]. Because, in the first layer of systems thinking, the system is seen as something real, or soon to be realized, there is limited rethinking of the perspectives from which a system’s elements, relationships, boundaries and emergent properties might be viewed [13]. Nevertheless, despite this limitation, it is still more insightful to see one’s own activities and those of others as part of an interactive, wider system than to view them all as isolated and independent. It is inadequate consideration of the wider system that leads to poor coordination between stakeholders and unanticipated side-effects of innovation.

The second layer involves *rethinking* elements of the system: its relationships, boundaries, emergent outcomes and/or the perspectives from which systems can be seen [4]. To rethink any or all of these requires *thinking about our thinking* (meta-cognition) and looking for the limitations of our current perspectives on the system. Once people understand the full implications of the idea that the system is always seen from a perspective, meaning that objectivity and comprehensiveness should not be assumed, the possibility that the system could be seen differently becomes easier to contemplate [9,13]. This second layer of systems thinking asks people to deliberately experiment with identifying and thinking about other possible relationships, boundaries, emergent outcomes and perspectives that might matter. This focus on rethinking systems adds extra complexity beyond the first layer, and learning it requires some practice. However, the second layer is well within the grasp of most people to use with a minimal investment of effort [15].

The third layer offers a collection of specialist systems methodologies and methods [8] that involve quite intensive systemic innovation processes, frequently in facilitated workshops and/or with expert analytic input. These processes have purposes that can be usefully pursued as part of systemic innovation [10], including, for instance, mapping complex causality to anticipate the likely knock-on effects of innovation, designing highly adaptive organizations at multiple scales, gaining better mutual understanding between stakeholders so they can agree on what innovations are required, and evaluating innovations once they have been implemented.

Historically, there has been relatively little communication between those researchers who only use the basic systems idea, those who talk about skills for rethinking systems, and those who have developed specialist methodologies. This is because they have been working in largely separate research communities with different research traditions. Indeed, many people based in just one of these three traditions either are unaware of the others or pay little attention to them. BeanMeals has forged all three traditions into a single systems thinking framework, showing how these traditions are complementary if viewed in terms of the three layers, and the new framework offers a wider range of options for supporting systemic innovation than any of the individual layers viewed in isolation.

The BeanMeals team were trained in and used all three layers to inform the ongoing development of its own action research. The first layer was easily appreciated by the team. Training in the second layer involved the team critiquing early assumptions about the project’s boundaries, relationships, perspectives and anticipated emergent outcomes. A workshop on the third layer was run to introduce the team to seven specialist systems methodologies and their associated methods, with an offer of follow-up support if/when a team member chose to use one. These seven methodologies were considered an adequate starting point for the team’s learning because they were designed for different purposes and had all been extensively tried and tested in practice. However, they were not presented as all that would ever be required: the likelihood of needing other methodological resources (including more traditional methods from the social and natural sciences) or developing something entirely new was explicitly discussed.

The team did indeed develop a new food system innovation methodology focused on trade-offs between economic, health, social and environmental outcomes in different UK bean production and consumption scenarios. An initial trade-off assessment was used as an input to enable a stakeholder dialogue around the desired directions for bean system (and wider food system) transformation (see §5).

In addition to the above training in the three layers of systems thinking, and development of a new specialist methodology, several planning and review workshops with the team and its key partners were facilitated using a mix of methods drawn from the methodologies offered as part of the third layer. This enabled mutual appreciation and learning between people with different disciplinary backgrounds, and it stimulated creative and critical engagement with the ongoing work of project design, beyond what might have been expected from traditional team meetings. Thus, the team undertook systemic innovation itself, in addition to facilitating systemic innovation by stakeholders and communities. Both proved insightful.

Over the coming pages, as different aspects of the BeanMeals project are introduced, there will be frequent references back to these three layers of systems thinking, with examples of how they manifested in the work of the action research team and project participants.

## 3. Promoting healthy diets with bean-based meals low in fat, salt and sugar

### (a) Introduction

Poor diet is a major contributor to non-communicable diseases, which highlights the need for equitable approaches to promoting dietary changes that target the food environment and related infrastructure [16]. Food preferences develop early in life and track into adulthood [17], so it is critical to create supportive environments for children to repeatedly taste novel foods and flavours [18]. Schools provide a significant setting for such efforts, given that almost 9.1 million children in England attend school, with 24.6% eligible for free school meals [19].

### (b) Action research in schools and homes

A significant portion of the BeanMeals action research centred on six primary schools and ten households in Leicester and Leicestershire, representing urban and rural wards in areas of deprivation that are ethnically and culturally diverse. The key aim was to understand how to innovate in school catering and home cooking to promote healthy diets by introducing bean-based meals using the new URBean varieties.

The school-based activities comprised new bean-based recipes introduced to lunchtime menus in the six schools between February and July 2023. The school cooks were provided with a supply of URBeans to integrate into their existing lunchtime menus. Food for Life (a schools programme run by the Soil Association) developed a bespoke, bean-focused, classroom curriculum aimed at children in year 5 (9- and 10-year-olds), which comprised lessons on growing, cooking and exploring tastes and textures of different beans. This was delivered alongside training to support the active involvement of teachers, lunchtime supervisors and school cooks.

A professional games designer also worked with children in a fun and engaging way to co-design ‘Beantopia’, a board game following the journey to the plate of the Godiva and Capulet beans. In addition to being a vehicle for learning about beans, the game provided a means for the research team to engage with the perspectives of children in a much richer way than a typical interview or group discussion.

Alongside the work in schools, ten households representing diverse backgrounds, including those experiencing challenging social and economic circumstances (e.g. precarious accommodation; single-parent households, often with large families; physical and mental health issues), were recruited from two of the schools. While this was a relatively small subset of the number of families engaged with, it enabled the researchers to see, in depth, what systemic innovations would be possible and how they might be perceived. Families attended three ‘Cook & Eat’ sessions, which provided them with opportunities for family members to cook together using the beans. Recipes and ingredients were taken home. Two visits (incorporating household-led tours of the kitchen, conversational interviews and video recordings of home cooking [20]) were made to each household to enable observations of the barriers and facilitators that families faced when incorporating UK-grown dried beans into their meals.

The decision to widen the boundary from schools to homes involved deliberate, second-layer systems thinking by the team (rethinking elements of the system): the original boundary defining the schools focus was critiqued on the grounds that what happened at school would interact with what happened in the home, and in some cases the sustainability of the school learning about beans could be undermined if it was not supported by parents and siblings.

As is usual in systemic innovation [2], the work within the schools involved collaboration between multiple actors, including pupils, teachers, headteachers, school cooks, lunchtime supervisors, parents, other members of the household, school caterers and Food for Life staff. The researchers viewed both the school and the home as an ecosystem (a metaphor that is commonly used as part of the first layer of systems thinking), which suggests that knowledge about using beans has to be developed in the context of family and school relationships. An example was discussion with parents about how to handle their children’s resistance to trying new foods. This was therefore not just innovation in cooking, but also innovation in the family relationships and ways in which the families would talk about food and come to think about the beans.

School and household cooks were both able to innovate within their parts of the food system to incorporate beans into their familiar and favourite recipes, and in some cases adopt new recipes. School cooks offered the beans in the mainly vegetarian lunchtime meal setting as composite meals (e.g. cottage pie, curry, chilli, pizza) and sides (e.g. homemade baked beans), with visible (e.g. beans identifiable in curry) and invisible (e.g. beans blended into pasta/pizza sauce) offerings. Household cooks creatively adapted recipes to incorporate mashed beans in Bolognese or pizza sauce, tomato-ey beany cheese panini and bean burgers.

### (c) Policy challenges

With schools being part of the public procurement system, they provided a vehicle to engage with local authorities, catering companies and suppliers as part of the action research and systemic innovation analysis. Public procurement of school food is a market driver that could offer a protected advance price to farmers, encouraging them to grow the beans, and generating space for new enterprises to emerge to facilitate bean processing and distribution, with health and environmental benefits as well as economic ones [11].

The BeanMeals policy analysis showed that national policies have cast shadows over public procurement and the quality of school meals. The principle of compulsory competitive tendering introduced in the 1988 Local Government Act emphasized best value in deciding local authority catering contracts, which has been interpreted as weakening quality criteria and reducing costs. While there are now positive signs that there is a move back to higher-quality food in school meals policy, tighter public budgets are creating counter pressures on schools. The marketization of education, and the move away from local authority provision of State school education to allow individual schools to take control of their own budgets as academies, and as members of multi-academy trusts, have made school meal provision more complex and diverse.

The national policy framework shapes local authority and school-level implementation of school meals policy, as well as the public procurement of food, across a dispersed and complex policy landscape. There are encouraging if somewhat disconnected signs of initial policy actions to promote greater direction and innovation in England’s school meals provision. DEFRA updated the Government Buying Standards for Food and Catering (GBSFC) for nutrition in 2021 to recommend a pulse-based main meal at least once a week in government institutions. However, the GBSFC only recommends ‘best practice’ for schools, so it is not mandatory [21]. The current proposed update includes a target for 50% of the public procurement spend to favour UK and locally grown sustainable produce, but while the consultation phase closed in 2022, no further policy has yet emerged [22].

The Procurement Act of October 2023, post Brexit, aims to make the UK procurement system more straightforward, encouraging decisions to be based on better quality, not just lowest cost [23]. A new online national platform for public sector catering procurement, *Buying better food and drink*, which went live in 2024, also promises to make it easier for local authorities to source from any preferred supplier approved on the new system [24]. The Department for Education has directed the school standards inspection agency, Ofsted, to play a stronger role in monitoring a school’s food culture and an ethos of healthy eating [25].

The rather dispersed and start–stop national policy approach, the reform of which was placed beyond the boundaries of this action research, underlines the need for systemic innovation in future policy making. This would involve greater coordination, dialogue and collaboration among the various government bodies, both national and local, involving school governors and heads, and reaching out to the chef and supervision levels in school canteens. If pursued, this would be an example of working with the first and second layers of systems thinking (possibly augmented with specialist methodologies from the third layer): seeking an enabling coherence across the wider policy landscape, involving key stakeholders at all levels in a coordinated change programme. Even in the absence of such a systemic innovation initiative, many examples of good practice in school meals delivery exist (for example, the Food for Life programme). However, for local authorities and their school meals providers, budgetary shortfalls and rising input costs (e.g. energy) are restricting what can be purchased and therefore the quality of the meals that can be offered. In this context, beans can offer a welcome, cheaper meal ingredient as a protein replacement for meat, and they can make a valuable contribution to children’s currently low vegetable intake, but there is a need for innovative recipes, presentation and supervision, as the action research illustrates.

## 4. How to produce and supply UK bean-based foods and ingredients

### (a) Agronomic, technical and policy challenges and opportunities; potential area and yield

Most beans consumed in the UK are an imported ingredient and sold as a processed food (tinned beans in tomato sauce). However, rather than replacing imported beans, the systemic innovation in BeanMeals defined the start-up marketplace for the domestic supply of URBean varieties as people cooking beans from scratch and those acting on nutritional health advice to increase human consumption of dietary fibre [26]. Production will focus initially in southern England (*ca* 158 000 ha below a line drawn from the Wash to the Severn Estuary), where the climate is best suited to launch and scale-up production [27]. This novel crop would be an alternative in arable rotation to pulses currently grown for animal feed, as both URBeans and animal-feed pulses share similar agronomy and are harvestable using a conventional combine harvester.

UK Government payments to farmers in England are directed to a range of incentives being offered under the Environmental Land Management Schemes (ELMS). To date, under ELMS and its Sustainable Farming Incentive (SFI) actions, there have been payments for planting non-food legumes as part of a farm’s nitrogen management for improved grassland, and as a beneficial fallow crop in rotations [28]. UK farmers collectively argue that complementary land management policy is needed for domestic food production, including food pulses for animal and human consumption (interview with Processors and Growers Research Organization (PGRO), 4 June 2024). There is some government funding for innovation in farm carbon management that encompasses the growing of animal feed beans, but not currently beans for human food [29]. Farmers lack two key pull factors to incentivize growing common or food beans: first, Government-directed initiatives; and second, a market price point equivalent to the current large commodity crops.

### (b) Demand scenarios

Initially, BeanMeals calculated the current *per capita* consumption of beans in the UK and used this as a baseline from which to plot potential increases in demand. The Family Food dataset encompasses both household and eating-out food purchases. Household purchases include canned peas, baked beans in sauce, other canned beans and pulses, dried pulses (excluding air-dried), soya and novel protein foods. Eating-out categories include baked beans and other beans and pulses (excluding green beans). Typically, beans absorb approximately their own dry weight of water during cooking, so the weight of cooked beans was halved to estimate their dry weight. For 'baked beans in sauce’, a conversion factor of 0.263 was applied to calculate the dry weight of beans, based on an experiment involving draining the tomato sauce and weighing the beans alone. Following this dry weight adjustment, data from the most recent 3 years (2021−2023) available in the Family Food dataset was averaged, producing an estimate of the mean consumption of beans among British individuals, which is approximately 8.5 g per person per day [30]. This level of intake is substantially below the recommendations in the Eatwell and EAT–Lancet dietary guidelines, underscoring a significant nutritional gap that necessitates strategic intervention to enhance public health outcomes.

Having estimated the consumption per person per day, the team constructed three stepwise scenarios of increased bean consumption based on different dietary guidelines, to estimate the changes in emergent food system outcomes that upscaling beans consumption from the baseline may produce.

The ‘Beans is How’ campaign aspires to double the current level of bean consumption globally by 2028 [31]. It advocates for the increased incorporation of diverse bean varieties into everyday meals, emphasizing their nutritional benefits and culinary versatility. Although ‘Beans is How’ has a higher *global* target, BeanMeals was only dealing with the UK. Therefore, the first scenario (S1) was set for 17 g per person per day: i.e. twice the current 8.5 g of beans per person per day.

The UK *Eatwell guide* [32] recommends a daily intake of 80 g of cooked legumes. Based on the Diet Impact Analysis toolkit developed by the World Health Organization [33], the 80 g of cooked legumes are converted to 34 g dry weight for the second scenario (S2).

The EAT–Lancet Commission’s guidelines [34] for a sustainable and health-promoting diet recommend the consumption of 50 g of dried beans, lentils and/or peas per day. This formed the third scenario (S3). While UK demand for plant-based foods is on the rise, it is concentrated around highly processed alternatives to meat rather than demand for minimally processed beans and legumes [35]. Despite the three demand scenarios discussed above, there is little evidence of a rapid increase in demand for minimally processed plant proteins, such as beans and other legumes. Further, while major caterers have a range of commitments related to plant-based foods on their menus, they do not have specific commitments around increasing demand for beans, let alone UK-produced beans. It may be that an increase in demand for beans is implicit in their plant-based targets, but this remains an assumption to be tested [36].

### (c) Enterprise opportunities

For demand to move towards increasing consumption of UK-grown beans, viable pathways for upscaling will be required. BeanMeals researched three potential enterprise pathways that were defined in terms of their scale and access to capital. These pathways were given the shorthand names ‘Community Enterprise’, ‘Artisanal Entrepreneurs’ and ‘Food Giants’. Each pathway involves different kinds of organizations with different capabilities, and each will have different implications for food system outcomes [36].

#### (i) Community enterprise

Community enterprise (pathway 1) is based on a closed-loop, short supply chain business model, using feedback from health-minded cooks in households and food services who prepare food from scratch in kitchens. The application of layer 1 systems thinking (seeing things as parts of real-world systems) helped the team identify the key positioning of cooks: they give rise to the initial demand for novel ingredients such as URBeans, and act as important agents of change for ‘fork to farm’ environmental and public health impacts. Using layer 2 thinking (rethinking the system), it became apparent to the team, through the rethinking of supply chain relationships and the boundaries of inclusion and exclusion, that a group purchasing organizer (GPO) is needed to fill the ‘missing middle’ between buyers and sellers in this pathway, which importantly is community-minded to manage affordable access to the ingredients. BeanMeals also applied two layer 3 methods, beginning with ‘viable system modelling’ [37] to envision a community-level design for a marketplace for URBeans. Then ‘critical back-casting’ [38] was used to develop specifications for Nurtural Food [39] as a potential social enterprise that could manage and scale up the community-minded marketplace. For the current URBean varieties, the production and sale of dry beans from pathway 1 will be launched following the 2025 harvest.

#### (ii) Artisanal entrepreneurs (new branded food products)

Pathway 2 (artisanal entrepreneurs) is characterized by innovative, entrepreneurial businesses bringing new bean-based products to market from an existing supply chain of dry beans; for example, Bold Bean Co., a fast-growing UK start-up company selling a range of imported jarred products. Such companies focus on branding high-quality products featuring cooked beans that appeal to the ‘foodie’ market of relatively affluent individuals who may not be satisfied by products currently provided by mainstream food manufacturers. Sales can thus be launched via farmers' markets and online sales. Companies within this pathway act as a driving force of food product innovation and combine novel products with creative, aspirational branding to shift consumer perceptions of beans.

#### (iii) Food giants

Pathway 3 (food giants) taps into established, high-volume, low-diversity value chains for canned and processed foods, using existing, large-scale infrastructure (mainly owned by large corporate companies). This pathway, including Heinz and Princes factories in the UK, depends on a reliable, uninterrupted supply of imported dry beans and sauce ingredients. The consumer in this pathway is mass-market, driven by food that is familiar, accessible, affordable and provides healthy nutrition.

Based on these three possible pathways, or combinations of them, there is capacity to increase pulse crops (including beans, peas and lentils) in arable rotations from the current 5% share to up to 20%, leading to a corresponding increase in the amount available on the UK market. While this has clear potential to increase consumption and hence health-related outcomes for the UK population, the potential positive and negative environmental and economic impacts will depend on where and how the increase is achieved.

## 5. Health, environmental and economic benefits and trade-offs of scaling UK beans

### (a) Introduction

BeanMeals evaluated the potential nutrition/health, environmental and economic implications of the introduction into the UK food system (including upscaling and consumption) of the new UK-grown beans. The outcomes from this introduction will vary based on (i) the volume of beans consumed (see the demand scenarios S1—S3 above), (ii) how and where the beans are grown (supply scenarios), and (iii) how fork and farm are connected (see the three upscaling enterprise models). The health, environmental and economic implications are discussed separately below, and the team's work on this assumes it is actually possible to bridge the gap between current bean consumption and the recommended intake in the three dietary guidelines already mentioned. While beans can contribute to greater diversity in current protein intake, there is nevertheless still a significant consumer acceptance barrier to be overcome [40], and this needs to be acknowledged.

### (b) Health/nutrition implications

Whatever the scaling pathway discussed above, meals designed around the new protein- and dietary fibre-rich beans can substitute for less healthy ingredients in meals (e.g. high-fat meat), replace less healthy options, and also increase satiety [41], potentially reducing the desire for snacks. These new varieties also have a low glycaemic index (GI), making them potentially suitable for both specialist meals and milling into low-GI flour for use as an ingredient in ready-meals and desserts to help manage the burgeoning increase in, and costs of, type 2 diabetes.

Upscaling bean demand (assuming they are consumed) also impacts nutrient adequacy. The scenarios indicate improvements in several key nutritional metrics, such as fibre, folate, iron and potassium. Analysis also indicates that beans can play a critical role in complementing other protein sources, allowing a reduced dependence on animal proteins (even though BeanMeals has not assumed a replacement of meat by beans). This shift is reflected in the gradual increase in daily protein contributions from legumes across the scenarios, increasing from 4 g in S1 to 12 g in S3. Consequently, the recommended protein intake (50 g day^−1^, UK Eatwell) that can be supplied from legumes increases from 8% in S1 to 23% in S3.

Another important aspect is the impact on maintaining a population with a balanced energy intake. The scenario analyses indicate that the caloric contribution of beans to the total dietary energy intake would remain relatively low. Considering the additional nutrients provided by beans and their minimal caloric contribution, integrating more beans into daily consumption patterns could potentially help improve diets and support the population in achieving healthier body weights.

### (c) Environmental footprint/potential

All activities along the bean value chain can make a useful contribution to the UK’s net-zero policy on greenhouse gas emissions [6]. BeanMeals compared the environmental impact of beans sourced from North America with those from the BeanMeals trial farm in Lincolnshire. Environmental indicators related to clean air and water, preservation of natural resources, biodiversity conservation and climate stabilization were used. A key assumption was that the three increased consumption scenarios (mentioned earlier) were to be met using beans entirely grown in the UK.

First, compared with North American production, growing beans in the UK does not require phosphorus fertilizer, and nitrogen fertilizer inputs are relatively low, thereby reducing phosphorus surplus in the soil and lowering nitrogen emissions to the air and water. Second, bean cultivation in the UK uses fewer herbicide varieties, reducing the risk of groundwater contamination and ecotoxicity. However, it is worth noting that the herbicides used on the BeanMeals test farm in Lincolnshire exhibit higher soil adhesion and persistence. This suggests that if these herbicides continue to be used in larger-scale production, there may be a higher risk of terrestrial acidification and ecotoxicity compared with production practices in North America. Third, although the global warming potential (GWP) of growing beans in the UK is slightly higher than in North America, this is outweighed by removing shipping emissions.

However, these benefits must be weighed against the challenge of allocating land in the UK for bean cultivation without displacing other crops. The complexity of land allocation highlights the need for balanced strategies that optimize local production benefits while ensuring sustainable land use and environmental stewardship.

### (d) Economic impacts

Assessing the economic aspects is challenging owing to numerous uncertainties related to the scaling-up of bean production, use and consumption. Also, it is unlikely that the UK could source all the necessary additional beans from home-grown production if the largest increase in consumption (recommended by the EAT–Lancet Commission) were to be realized. The economics are further complicated by the inclusion of increased North American imports.

Imported navy beans from North America cost around £1000 t^−1^. This sets a minimum farmgate value for developing a robust supply chain that offers UK farmers equitable financial incentives to cultivate URBeans. Such a supply chain also needs to ensure affordable access to the beans as a food ingredient for UK consumers. Thus, a transformative marketplace for higher-value domestic bean production needs to couple public health messaging (e.g. increasing UK consumption of dietary fibre) with UK farm benefits (e.g. improving soil health management), while adding supply options to essential imported beans.

Clearly, growing UK beans would help the UK balance of payments, as well as boosting UK farmer incomes, if the above conditions for viability can be met. However, as mentioned earlier, the challenge lies in identifying suitable land with suitable weather conditions within the UK for bean cultivation.

In terms of affordability for the consumer, the potential change in daily dietary costs resulting from increased bean consumption, but assuming there is no other change to diet, is minimal, rising by only £0.02, £0.06 and £0.09 (UK pounds) for scenarios S1, S2 and S3, respectively.

### (e) Trade-off assessment

Trade-off assessments are often complicated by insufficient clarity on exactly what needs to be transformed. The implication is that it is the food system’s health, environmental and economic outcomes that need to be improved (i.e. transformed) from suboptimal to more optimal [42]. But it involves ensuring not only better individual outcomes, but also a better balance amongst them, i.e. the acceptability of several trade-offs.

BeanMeals proposed a new food system innovation methodology to add to the set of specialist methodologies and methods already available in the third layer of systems thinking. The purpose of this was to inform stakeholder discussions of directions for change of the wider envisioned food system. Discussions tackled the question of how to manage potential trade-offs *and* anticipated synergies: it should not be assumed that every ‘win’ is accompanied by a ‘loss’ somewhere else—often, gains in one area can be synergistically combined with gains elsewhere to create ‘win–win’ outcomes [43], and sometimes achievement of one pivotal gain can make the achievement of subsequent gains easier to realize. The project developed and implemented this methodology, and the analyses indicated that, while overall benefits to health could be achieved, benefits to the environment mainly depend on the methods of land conversion and weed management used, and economic benefits (which could be realized by different stakeholders in different locations over different timescales) depend principally on the scaling model employed.

There is also the difficult question of how to bring dissenters along with a change that everyone else sees as beneficial. A way forward here is high-quality dialogue, helping everyone to develop a more nuanced understanding of the complexities [44]. This can support collective, creative thinking about ways to address and mitigate trade-offs. Thus, stakeholder learning is facilitated, and the trade-offs are not framed as ‘wins’ and ‘losses’. Paradoxically, this means not using the concept of trade-offs up-front with stakeholders to frame the dialogue, even if researchers understand what is happening as a trade-off process, because the term ‘trade-offs’ can be misunderstood as win–lose only and entrench conflict. Rather, the dialogue needs to be framed as an exploratory learning process where people gain better mutual understanding of the perspectives in the room and an improved appreciation of the complexity they are experiencing, and can search for creative solutions to the dilemmas they are grappling with.

## 6. Conclusion

The BeanMeals analysis has focused on the UK bean system, revealing where opportunities arise and where potential stumbling blocks and stakeholder push-back might come from. Potential clearly exists for increased demand to be triggered, and a more coherent governmental policy direction. Future systemic innovations within the food system could involve making beans available in easy-to-use, sustainable and low-cost formats that are appealing to children and families (e.g. beans incorporated into familiar and well liked foods). Also, innovations that bypass the need for soaking and/or boiling within the kitchen would be welcome: even though these bean varieties need less soaking from dried than many others, the pre-preparation and time required for this was noted by some of the schools and families as a barrier to regular use.

Potential also exists for increased production, albeit from a very low baseline, if benefits to farmers can be made clear. But this will also need investment in supply infrastructure, depending on how demand develops (whole beans, dried/canned, processed composite products, protein-based products, etc.).

The obvious potential is to deliver systemic innovations that work across the whole bean value chain to ‘transform the system’. The three layers of systems thinking can usefully support this. Systemic analysis in the context of action research, of the kind undertaken in BeanMeals, could also be useful in the wider debate on food system transformation, as it helps to enable a discussion across food system actors on the overall direction of change, and how to manage trade-offs and the ‘costs of change’ along the way. BeanMeals has developed a new food system innovation methodology for approaching trade-offs and the direction of change, firmly anchored in the practice of constructive dialogue, and this has the potential for further application and development through an iterative methodology–practice learning cycle.

## Data Availability

This article has no additional data.
